# Laryngomalacia and Obstructive Sleep Apnea in Children: From Diagnosis to Treatment

**DOI:** 10.3390/children11030284

**Published:** 2024-02-25

**Authors:** Luca Cerritelli, Andrea Migliorelli, Alessio Larini, Andrea Catalano, Alberto Caranti, Chiara Bianchini, Andrea Ciorba, Francesco Stomeo, Claudio Vicini, Stefano Pelucchi

**Affiliations:** 1 ENT & Audiology Unit, Department of Neurosciences, University Hospital of Ferrara, 44100 Ferrara, Italy; l.cerrittelli@ospfe.it (L.C.); mglndr1@unife.it (A.M.); alessio.larini@edu.unife.it (A.L.); ctlndr@unife.it (A.C.); crnlrt@unife.it (A.C.); chiara.bianchini@unife.it (C.B.); francesco.stomeo@unife.it (F.S.); pls@unife.it (S.P.); 2 Gruppo Otorinolaringoiatrico della Romagna, Primus Medical Center (GVM), Via Punta di Ferro, 2/c, 47122 Forlì, Italy; claudio.vicini@unife.it

**Keywords:** laryngomalacia, obstructive sleep apnea, OSA, DISE, children, diagnosis

## Abstract

The aim of this review is to investigate the state of the art among the association between Obstructive sleep apnea (OSA) and laryngomalacia, analyzing the epidemiology, the diagnostic tools, and the possible treatments available to affected patients. Laryngomalacia, characterized by the malacic consistency of the epiglottis with a tendency to collapse during inspiratory acts, producing a characteristic noise known as stridor, is a common condition in infants and particularly in those affected by prematurity, genetic diseases, craniofacial anomalies, and neurological problems. Congenital laryngomalacia, presenting with stridor within the first 15 days of life, is often self-limiting and tends to resolve by 24 months. OSA is not only a consequence of laryngomalacia but also exacerbates and perpetuates the condition. Currently, the treatments reported in the literature are based (i) on medical therapies (including watchful waiting) and (ii) on surgical treatments. Among the surgical techniques, the most described is supraglottoplasty, performed with the use of cold instruments, CO_2_ LASER, transoral robotic surgery, or the microdebrider.

## 1. Introduction

Laryngomalacia is a common condition in infants characterized by a malacic consistency of the epiglottis with a tendency to collapse inward during inspiration, producing a distinctive noise known as stridor. Its pathogenesis remains controversial, with several contributing factors [1].

Congenital laryngomalacia, which presents with stridor within the first 15 days of life, is often self-limiting and tends to resolve by 24 months of age. Stridor is often triggered by feeding, crying, in the supine position, or distress and may be associated with feeding difficulties, coughing, choking, and slow feeding [2].

Late-onset laryngomalacia may manifest without the above symptoms, with stridor only occurring during physical activity or during sleep; in this case, it is defined as occult laryngomalacia [3]. The clinical problem of laryngomalacia is the airway collapse during breathing, especially at night, leading to stridor and obstructive sleep apnea.

The association between laryngomalacia and Obstructive sleep apnea (OSA) has been described among the infant population: in these patients, laryngomalacia is initially diagnosed using stridor, and subsequently, polysomnography is performed to exclude obstructive sleep apnea and to refer the patient for treatment. In contrast, older children are first identified by their symptoms of sleep-disordered breathing (snoring, hyperactivity, etc.) and are often surgically treated using adenotonsillectomy, with or without preoperative polysomnography [4].

Sleep apneas are not only a consequence of laryngomalacia but also exacerbate and perpetuate the condition. Increased inspiratory resistances generate higher intrathoracic negative pressures, which can worsen gastroesophageal reflux, further altering epiglottis conformation, escalating inspiratory resistance, and therefore creating a vicious cycle. The degree and severity of laryngomalacia are related to the presence of associated symptoms and not to the stridor frequency or loudness [5]. Mild laryngomalacia is characterized by inspiratory stridor without other symptoms; moderate laryngomalacia is associated with cough, regurgitation, choking, and feeding difficulties. Severe laryngomalacia may be associated with apneic episodes and cyanosis, growth failure, pectus excavatum, pulmonary hypertension, and cor pulmonale [6]. In particular, growth failure has been related to breathing and feeding; the increased metabolic intake necessary to coordinate these actions can be so severe that both can be somehow impaired, leading to weight loss [7]. Prematurity, genetic diseases (syndromic like Down’s syndrome), craniofacial anomalies, and neurological problems (such as hypotonia) can often be associated with OSA and laryngomalacia [8,9,10,11,12].

The benefit of reducing OSA is an important issue to consider. Although laryngomalacia is usually a self-limiting condition, many significant short and long-term consequences may appear as the consequence of intermittent hypoxia, frequent arousals, and sleep fragmentation; several studies have suggested that OSA has a deleterious effect on infants’ mood and possibly on their intellectual development [13]. In fact, the ongoing development of the neocortex and specialized brain pathways is critical during infancy [13]. In children with OSA, the gold standard treatment is surgical adenotonsillectomy [11]. However, laryngomalacia and tongue base collapse have been shown to be major causes of persistent OSA after adenotonsillectomy in children by drug-induced sleep endoscopy (DISE) studies [14]. Consequently, surgical correction using supraglottoplasty techniques has been proposed in these cases, performed with the use of CO_2_ lasers, transoral robotic techniques, or microdebriders. Syndromic children with neurologic anomalies and laryngomalacia have been reported to be at higher risk for treatment failure, with higher rates of revision surgery (47.8% vs. 18.2%) and tracheostomy (39.1% vs. 0.0%) [15].

The aim of this review is to investigate the state of the art concerning the association between OSA and laryngomalacia, analyzing the epidemiology, the diagnostic tools, and the possible treatments available to affected patients.

## 2. Methods

The present is a narrative review of the English literature on laryngomalacia and OSA. It has been performed using Medline, PubMed, Google Scholar, and Scopus databases. Two searches have been performed using the keywords “Laryngomalacia OSA” and “Laryngomalacia obstructive sleep apnea”.

Papers focusing exclusively on syndromic and genetic causes of laryngomalacia and OSA have not been included.

## 3. Results and Discussion

A total of 130 relevant articles on this topic have been identified by the search. Only English-language articles published between January 2000 and November 2024 were included.

Furthermore, we considered papers in which only children were analyzed.

Articles without abstracts or with missing data were excluded.

Finally, a total of twenty-one articles were selected after title and abstract screening, of which 16 were included upon completion of the full-text review [3,4,16,17,18,19,20,21,22,23,24,25,26,27,28,29].

In infants, laryngomalacia is one of the major causes of OSA, along with adenoid hypertrophy, nasal airway obstruction, and syndromic skeletal anomalies, as shown in Kaditis’ review [30]. The natural course of a child with laryngomalacia and OSA is unknown. In many cases, symptoms, including stridor, may resolve within 6–8 months; however, in others, apneas may persist despite the resolution of the stridor. The prevalence of OSA in patients with laryngomalacia varies between studies, ranging from 3% to 77% [24,31,32,33]. Previous meta-analyses have investigated the results of surgery in children with OSA and laryngomalacia, and a reduction in the apnea-hypopnea index (AHI) and of the oxygen desaturation index (ODI) after surgery has been reported.

### 3.1. Epidemiologic Features

OSA is one of the most common health problems in the pediatric population, affecting 1% to 4% of all children in the United States [29]. The peak incidence of OSA in children is reported to range between 2 and 8 years due to the increased size of the lymphoid tissues within this age [34]. Nevertheless, the incidence and causes of OSA in young infants under 2 years of age are still understudied, as the diagnosis of OSA in this age group is very challenging due to the limitations of polysomnographic techniques [35]. The percentage of children with OSA and laryngomalacia varies in different studies, ranging from 79% to 14.3% [14,36] in relation to the children’s age.

### 3.2. Etiologic Theories and Risk Factors

Currently, there are several etiologic theories of laryngomalacia in the literature, but the precise etiopathogenetic features remain controversial [1]. Proposed etiologies include the anatomic theory, the cartilaginous theory, the neurologic theory, or a combination of these [3,10,37,38,39] (Table 1).

The “anatomic theory” is based on the evidence that one of the anatomic abnormalities seen in laryngomalacia is an unusually long and flaccid epiglottis, omega-shaped, which is displaced posteriorly against the posterior pharyngeal wall during inspiration. Another possible finding is the presence of short aryepiglottic folds that may retroflex a normally shaped epiglottis [3]. In other cases, bulky arytenoids may prolapse anteriorly during inspiration due to redundant mucosa overlying the arytenoids [40]. These changes may occur separately or in combination, and the most common is the association of a shortened aryepiglottic wall and a redundant arytenoid mucosa [16]. Malformations of the upper airway, of the central nervous system [41], and/or of the cardiac system may also be associated.

The “cartilaginous theory” refers to disorders in the cartilaginous framework of the larynx and trachea, causing a greater laxity in supraglottic structures [37]; a particular cartilage ‘immaturity’ with an increased malleability has been proposed by histological studies [1,42]. Gastroesophageal reflux (GER) is an important cofactor, probably causing cartilaginous modification and edema of the laryngeal mucosa, with a consequent obstruction of the laryngeal lumen [43].

Matthews et al. [43], with double probe pH monitoring, demonstrated that most children with laryngomalacia have a larynx exposed to acid on a regular basis. Garritano et al. [44] found GER in 88.2% of young patients undergoing supraglottoplasty for laryngomalacia and reported that gastroesophageal reflux and apnea may have a close relationship [38]. Arad-Cohen et al. [45] reported that apnea preceded reflux in 93.6% of episodes, and only 6.4% of apneic episodes were followed by reflux. Menon and colleagues [46] reported an increased frequency of apnea in infants with regurgitation, but this was not related to GER, while Kamal et al. [47] recently reported that neonates affected by GER developed OSA.

It is likely that laryngomalacia is often associated with GER [48]; however, the role of GER in the pathogenesis of laryngomalacia remains controversial. Even if several studies [1,42] have highlighted the presence of mild inflammation in the surgical specimens of children who undergo supraglottoplasty for laryngomalacia, eosinophilia, a proposed histological marker of GER [42], was only identified in a few cases (3 out 61). Evidence supporting the use of acid suppression therapy (AST) for laryngomalacia is limited because of the lack of randomized controlled trials [49].

The “neurological theory” [10] is supported by the fact that a child with laryngomalacia often has neurological comorbidities (with an incidence of 8–50%) correlated with hypotonia, prematurity, a history of Apparent Life-Threatening Event (ALTE). These children also have congenital syndromes (18.5%), with genetic anomalies present in 8–20% of cases, mainly represented by Down syndrome. Tanphaichitr and colleagues [50] have highlighted that children with laryngomalacia have a high incidence of central sleep apnea (CSA), demonstrating altered laryngeal tone and sensorimotor integrative function of the larynx, with subsequent lack of neuromotor coordination [39]. Weak laryngeal tone, apnea, and swallowing problems often coexist with laryngomalacia and could be related to an abnormal function of the pathway at the brainstem nuclei. The tendency for this condition to improve spontaneously within the first two years of life provides further support that growth and maturation may reduce the collapsibility of supraglottic tissues.

Obesity has been investigated as a potential link between laryngomalacia and obstructive apneas by Kennedy et al. [27], with a 578.1% increase in the frequency of laryngomalacia in patients with obesity compared to patients without obesity. Obese individuals have a greater potential for laryngeal collapse, particularly due to a high concentration of adipose tissue around the head and neck [51].

Children with laryngomalacia could present Synchronous Airway Lesions (SALs), with an incidence of 7.7–51.7% [52,53]. Bredun and colleagues [54] have described these lesions in 15.1% of children with laryngomalacia, and the associated malformation consisted of laryngotracheoesophageal cleft (61.1%), tracheomalacia and tracheobronchomalacia (16.7%), trachea-esophageal fistula (5.6%). Therefore, the endoscopic evaluation of laryngomalacia should always include the assessment of the lower airways in order to consider the eventual presence of SALs that could have a further clinical impact [55].

### 3.3. Diagnosis

The diagnostic suspicion of laryngomalacia in infants (<2 years of age) arises from inspiratory stridor and can be easily confirmed by awake rhinopharyngolaryngoscopy. This diagnostic method allows us to appreciate static and dynamic anatomical supraglottic anomalies.

Several endoscopic classification systems have been proposed, firstly by McSwiney [56]. The most recent and simple, the Groningen classification of laryngomalacia [57], distinguished three different types of laryngomalacia: Type 1: the inward collapse of the arytenoid cartilages; Type 2: medial displacement of the aryepiglottic folds; and Type 3: posterocaudal displacement of the epiglottis against the posterior pharyngeal wall.

Rhinopharyngolaryngoscopy is also a critical procedure to rule out other potential anatomic airway lesions, such as laryngeal clefts or superior subglottic stenosis (5.6–16.7%) [25].

The diagnosis should be made as early as possible and eventually on the first day of life if stridor is present.

In older children, laryngomalacia should also be suspected in cases of obstructive sleep apnea symptoms, such as snoring, hyperactivity disorders, or attention-deficit.

In children even older, laryngomalacia should be suspected in case of recurrent OSA after adenotonsillectomy. Therefore, polysomnography (PSG) is essential to document OSA persistence or recurrence, and the International Pediatric Otorhinolaryngologic Group (IPOG) [58] has recently recommended PSG in the diagnostic work-up and decision-making in laryngomalacia, particularly when significant apnea is present.

DISE, which consists of the use of flexible fiberoptic rhinopharyngolaryngoscopy under sedation in the operating room with the patient spontaneously breathing, is useful in identifying potential surgical targets in refractory cases. Hypertrophy of the lingual tonsils and laryngomalacia are the most common findings in DISE studies [28].

Under sedation, it is also possible to complete the airway examination using a microlaryngotracheobronchoscopy, including the tracheal segment of the airway. This procedure can rule out the presence of tracheomalacia or subglottic stenosis, which is also possibly associated with laryngomalacia [59].

Sleep endoscopy is a novel diagnostic tool in the evaluation of children with obstructive sleep apnea that may lead to the observational diagnosis of sleep-dependent laryngomalacia in the absence of other symptoms or daily stridor [20].

Comparison between rhinopharyngoscopy and DISE is difficult as the airway dynamics differ between awake and asleep statuses and since each technique has specific inherent limitations [28]. Therefore, it may be reasonable to consider fiberoptic evaluation and DISE as complementary modalities when assessing supraglottic airway dynamics and severity of obstruction in infants with laryngomalacia, especially if surgical treatment is needed. Boudewyns et al. [60] showed that upper airway evaluation using DISE changed the treatment decision in 1/3 of their infants less than 2 years of age. This finding suggests that 1/3 of their patients could have been treated inadequately with standard adenotonsillectomy, and the multilevel obstruction could have been missed. Digoy and colleagues [26] described the procedure in children with laryngomalacia to evaluate the laryngeal contribution to sleep apnea. They performed the procedure under general anesthesia, induced with sevoflurane (8%) in 100% oxygen. The administration of sevoflurane was then reduced or stopped while the patient was spontaneously breathing, and at that moment, the surgeon performed either indirect laryngoscopy or direct laryngoscopy. Occasionally, small doses of intravenous propofol were administered to perform direct laryngoscopy. As the child then neared stage II anesthesia, dynamic breathing and pharyngolaryngeal muscle movements were examined. Therefore, they modified the practice in favor of flexible endoscopy, as this method can be done at a lighter level of anesthesia and may provide a more valid assessment of airway dynamics (see also Table 2).

At present, there is no unanimous consensus in the literature on DISE procedures in patients with laryngomalacia. Often, the assessment is performed under sedation in direct laryngoscopy, with the patient still breathing spontaneously; in other cases, a flexible instrument is used with different drugs for the sedation. Several methods have included propofol alone [61], propofol/narcotic combination [12], dexmedetomidine, ketamine/dexmedetomidine, and midazolam/narcotic [62,63,64]. Additionally, the type of laryngeal alteration detected is not always reported. No universal classification is used, and a comparison of results between different studies is difficult.

Some authors suggested the use of bronchoscopy to exclude the possible presence of associated subglottic malformations. Certainly, the use of this method is indicated when laryngeal endoscopic is negative, and this examination fails to fully explain the child’s symptoms [65].

**Table 2:** DISE: demographic and diagnostic features within the included studies.

Authors	Previous PSG	DISE	N	Male	Mean Age	Genetic Syndrome	R	SAF	BA	M
Love 2020 [66]	Yes	Yes: propofol + Sevoflorane	41	64.10%	11 mo.	22.00%	NA			
Bhushan 2019 [23]	Yes	Yes	41	53.60%	1.3 yrs.		NA			
Digoy 2012 [26]	Yes	Yes: laryngoscopy/flexible nasendoscopy via light general anesthesia (sleep endoscopy) sevoflurane (8%) in 100% oxygen	36		56 mo.	25.6%	NA			
Mase 2015 [20]	Yes	Yes: flexible nasendoscopy under total intravenous general anesthesia (propofol)	9	55.50%	17 mo.		NA			
Boudewyns 2017 [14]	Yes	Yes (no details)	28	60.7%%	1.5 yrs.		NA			
Chan 2012 [3]	Yes	Yes: flexible fiber-optic sleep endoscopy	22	73.00%	7.4 yrs.	27.00%				
Revell 2010 [4]	Yes	Yes: direct laryngoscopy under intravenous anesthesia (spontaneous ventilating)	51	50.90%	7.2 yrs.					
Garritano 2014 [45]	No	No: direct laryngoscopia previous supraglottoplasty surgery	17	64.70%	33.7 mo.	11.80%	94.10%	94.10%	29.40%	
Powitzky 2011 [19]	Yes	No: flexible laryngoscopy while inhaling Sevoflorane	20		3.9 mo.	15.00%				
O’Connor 2009 [18]	Yes	No: fiberoptic nasopharyngoscopy	10	70.00%	2.6 mo.	20.00%	40.00%	100.00%	90.00%	
Weinstein 2016 [67]	Yes	Unknown: fiberoptic nasopharyngoscopy AND direct laryngoscopy	23	69.50%	7.1 mo.					
Ching 2017 [21]	Yes	No: fiberoptic nasopharyngoscopy	8	62.50%	13.1 mo.					
Cortes 2019 [22]	Yes	No: fiberoptic nasopharyngoscopy OR direct laryngoscopy	9	55.50%	5.5 mo.		77.70%	88.80%	100.00%	
Fard 2020 [36]	Yes	No	108							
Vberkest 2020 [24]	Yes	No: fiberoptic nasopharyngoscopy AND direct laryngoscopy	44	54.50%		25.00%	2.00%	19.00%	25.00%	50.00%
Ratanakorn 2021 [68]	Yes	No: fiberoptic nasopharyngoscopy	57	47.30%	3.6 mo.					
Zafereo 2008 [17]	Yes	No: fiberoptic nasopharyngoscopy	10		4 mo.					
Valera 2006 [16]		No: fiberoptic nasopharyngoscopy AND direct laryngoscopy	7	57.10%	6.8 mo.		100.00%	100.00%	100.00%	

Legend: N: number of patients; mo.: months; yrs.: years; NA: no data available; R: Retropositioned/omega-shaped epiglottis; SAF: Short aryepiglottic folds; BA: Bulky arytenoides; M: Miscellaneous.

### 3.4. Treatment and Outcomes

Laryngomalacia most commonly affects infants and improves spontaneously by 2 years of age, as already described [69]. While in the majority of cases, laryngomalacia follows this safe pathway, rarely can it be severe enough to cause breathing and feeding disorders that can impact normal development and growth. In the most severe cases, laryngomalacia has been described as a cause of sudden infant death syndrome [70].

Approximately 5–20% of severe or refractory children require surgery [71]. To date, supraglottoplasty is the first-line treatment for this condition [5,25]. Surgical techniques to treat this pathology have evolved over time, ranging from tracheotomy to transoral procedures. Historically, tracheotomy was the only method of treating laryngomalacia [72]. The first surgical approach to epiglottis was performed by Iglauer in 1922, who removed the epiglottis using a nasal sling [73].

The use of supraglottoplasty for the treatment of severe laryngomalacia was first described by Holinger et al. in 1989 [72]. A year later, its use in the treatment of OSA in pediatric patients with laryngomalacia was described. Currently, this technique represents the gold standard for the management of this condition [74]. Supraglottoplasty can be bilateral or unilateral and can be performed with cold instruments, LASER CO_2_, debridement, or radiofrequency [3,16,17,18,19,20,21,22,23,24,25,26]. This technique includes several procedures that are performed depending on the pathological site found (e.g., shortened aryepiglottic wall, omega-shaped epiglottis, redundant mucosa of the arytenoid regions) and it is anatomically divided into epiglottoplasty, aryepiglotticoplasty, and arytenoidoplasty [75]. Therefore, the main principle is to treat only the altered supraglottic structures and to tailor the treatment for each patient [18].

**Figure 1:**
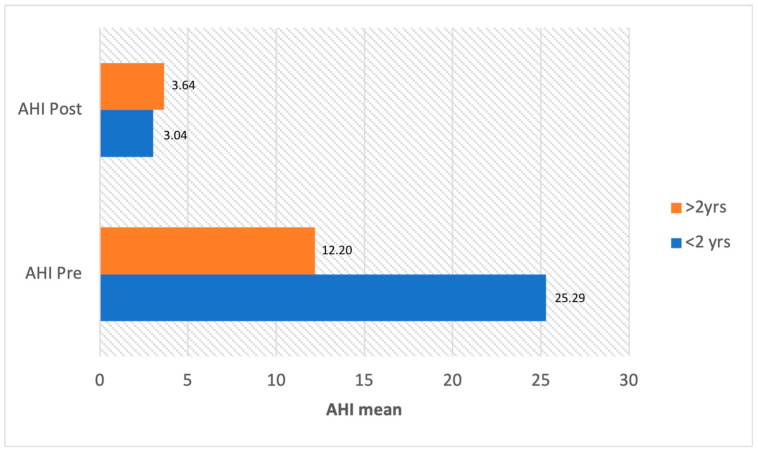
Average AHI before (AHI Pre) and after surgery (AHI Post) in literature patients divided by age.

Laryngomalacia can also occur in children over the age of 2, often in association with residual OSA following adenotonsillectomy [12,76,77]. Clearly, the clinical, diagnostic, and especially therapeutic indications differ significantly between birth-onset and late-onset laryngomalacia. Patients with congenital laryngomalacia usually have a higher average AHI than patients with late laryngomalacia, but supraglottoplasty leads to excellent postoperative results in both groups (Figure 1). Treatment features within the included studies are presented in Table 3.

#### 3.4.1. Population under 2 Years

**Figure 2:**
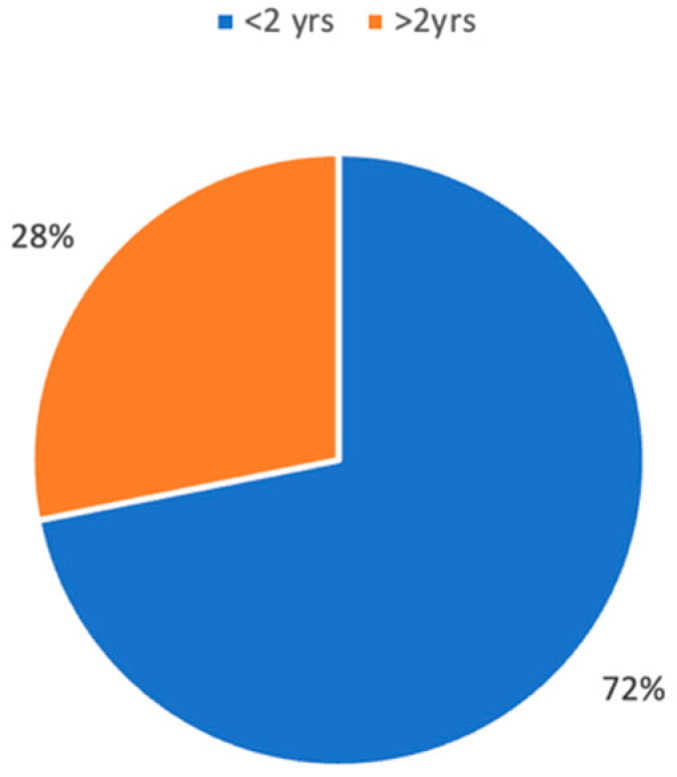
Percentage of patients evaluated in the literature by age.

Most patients studied in the literature are younger than two years of age (Figure 2). When analyzing congenital laryngomalacia, the majority of patients are treated with medical therapy or watchful waiting strategies. Many conservative treatments can be used, including nasal steroids and montelukast. Since the relationship between GER, laryngomalacia, and OSA has been documented in the literature, the use of antireflux medications (e.g., H2-BID blockers or proton pump inhibitors) is one of the cornerstones of medical therapy and is often used after surgery [25]. Other treatments, such as supplemental oxygen or breathing stimulants, may be indicated for children with OSA [39].

Medical management is always required in combination with surgery, and several medications have been proposed in combination; Powitzky et al. [19] suggested a twice-daily proton pump inhibitor after supraglottoplasty for at least one month postoperatively, while steroids were occasionally administered for less than 24 h postoperatively [44].

Surgery is indicated in cases of severe laryngomalacia. Laryngomalacia severity is not related to stridor intensity or frequency but to the presence of associated symptoms [5]. Currently, the surgical indications available in the literature are not clear and standardized but are often based on the experience of individual centers. The most common indications are severe dyspnea, poor development, psychomotor retardation, pulmonary hypertension, cyanosis, dysphagia, aspiration, and sleep apnea syndrome [16,17,18,19,20,21,22,23,24,25].

Furthermore, laryngomalacia may occur alone or in association with other comorbidities (e.g., GER, craniofacial anomalies, and neurocognitive disease), and the presence of the latter may determine a lower probability of surgical success can increase possible postoperative complications [71,78]. In cases of pharyngolaryngomalacia, tracheotomy may be indicated, given the poor results of supraglottoplasty [16].

In congenital laryngomalacia associated with OSA, supraglottoplasty is often the first-line treatment since adeno-tonsillar hypertrophy usually does not develop in children under 2 years of age. Over the past 20 years, an increasing number of studies have used PSG before and after surgery to evaluate its efficacy. In 2006, Valera et al. evaluated the results of supraglottoplasty using PSG in a sample of 7 OSA children with an average age of 7.14 months. The authors have concluded that supraglottoplasty resulted in significant PSG and symptom improvement in patients with laryngomalacia. In contrast, in patients with pharyngolaryngomalacia, supraglottoplasty does not produce positive results, and tracheotomy should be preferred [16]. Similar results were found by Zafereo and colleagues in 2008; in a sample of 10 patients with a mean age of 4 months, the mean AHI decreased from 12.2 to 4.2 events/hour at 11 weeks after surgery [17]. These results are confirmed and enhanced by an even greater reduction in AHI (42.7 preoperative vs. 4.47 postoperative) by O’Connor et al., with statistically significant improvements also found in mean total sleep time, lower oxygen saturation level, and respiratory distress index [18]. In these papers, the importance of PSG for both diagnosis and monitoring of supraglottic outcomes has been evidenced.

The three aforementioned papers all used cold surgical techniques. Powitzky et al. instead used CO_2_ LASER in a population of 20 neonates with a mean age of 3.9 months and found an improvement in AHI in all patients starting from a baseline score of 5 or greater. On the other hand, the authors describe a worsening of AHI in all patients with mild OSA because of subsequent adenotonsillar hypertrophy in this group [19]. In 2017, a unilateral supraglottic technique using bipolar radiofrequency ablation was described. Unilateral techniques were developed to avoid supraglottic stenosis, which, although rare, is a complication that can lead to tracheotomy and subsequent numerous revision surgeries. Again, the authors found a significant reduction in AHI from 19.3 to 4 events/hour [21].

Considering that laryngomalacia often resolves spontaneously within two years, it is reasonable to question whether the improvement seen in weeks or months after surgery is caused by the surgery itself or by the natural evolution of the pathology. In this way, an important study comparing the results of surgery with those of conservative therapy has been published recently. The authors compared 18 patients treated using supraglottoplasty and 12 patients treated using conservative therapy. The results show that surgery achieves a reduction in AHI of approximately 12.6 events per hour compared to a reduction of 3.3 events per hour achieved with medical therapy alone. Nevertheless, supraglottoplasty was not superior to medical therapy in a head-to-head comparison (*p* = 0.09). This work is limited by the small sample size. Furthermore, it should be highlighted that many patients treated conservatively had mild to moderate OSA, whereas those treated surgically had moderate to severe OSA [25].

According to the available data, supraglottoplasty appears to be a valid and effective technique for the treatment of severe laryngomalacia in OSA patients under two years of age. PSG assessment has now become essential to study patients with laryngomalacia and OSA and to have an objective method to evaluate surgical outcomes.

Supraglottoplasty is particularly indicated in the treatment of moderate to severe OSA; the greater the severity, the greater the benefit in terms of AHI reduction. In contrast, its role in the treatment of patients with mild OSA (AHI less than 5) is still unclear, and further studies will be needed to evaluate its efficacy. Finally, it will be important, in the future, to compare the outcome of surgical treatment vs. medical treatment, stratifying by severity of OSA and degree of laryngomalacia.

#### 3.4.2. Population over 2 Years

Poor somatic development and cardiovascular and neurocognitive outcomes are associated with untreated OSA in children. Adenotonsillar hypertrophy is the leading cause of OSA in the pediatric population, and adenotonsillectomy is the first-line treatment for these patients [79].

Several comorbidities, including obesity, asthma, and severe preoperative OSA, have been shown to increase the likelihood of residual OSA following adenotonsillectomy. Therefore, a significant number of these children will have other anatomical etiologies of OSA that can be surgically corrected [3].

Laryngomalacia is considered a risk factor for OSA and is one of the potential causes of residual OSA after adenotonsillectomy. This condition has been found in 3.9% of children with OSA [12]. In addition, stridor is often absent in these patients, and laryngomalacia is diagnosed late [3].

Supraglottoplasty is a viable treatment option for children with laryngomalacia and residual OSA. Chan et al. described their experience with 22 children who underwent supraglottoplasty between the ages of 2 and 18 years. All patients underwent preoperative and postoperative PSG and were evaluated using preoperative DISE. The surgical procedures were performed with CO_2_ LASER. None of the patients presented with stridor, feeding difficulties, or growth failure. PSG parameters improved in 91% of cases [3].

In 2012, a similar paper was published on 36 patients with a mean age of 4.5 years treated with supraglottoplasty for laryngomalacia and OSA. As before, pre- and postoperative PSG was used to assess the surgical outcomes, and DISE was performed to guide and plan the surgery. Again, the authors found a statistically significant improvement in AHI postoperatively [26].

Therefore, in children older than 2 years, with residual OSA after adenotonsillectomy or with small tonsils, PSG is essential for the assessment. DISE is also an important tool to identify possible sites and patterns of obstruction and to assess the presence of laryngomalacia. These tools are of paramount importance for planning subsequent surgery in children with residual OSA (tailored treatment).

#### 3.4.3. Complications and Follow-Up

Postoperative complications are rare and include extubation failure, lung collapse, dysphagia, granulomas, aspiration, and supraglottic stenosis [16,18]. Postoperative dysphagia is common but usually transient [26].

Supraglottic stenosis is more common with bilateral techniques, so unilateral techniques have been developed over the years; the disadvantage of the latter is the possible need for a re-intervention [21]. The most common revision procedures are tracheotomy (especially in the presence of pharyngolaryngomalacia), revision supraglottoplasty, and adenotonsillectomy [16,19]. The latter should be considered when persistent OSA is present after supraglottoplasty associated with adenotonsillar hypertrophy.

Children with hypotonia or neurologic disorders associated with congenital laryngomalacia have been described to have poorer outcomes [15], more often requiring additional surgery or tracheostomy, and have a significantly higher postoperative AHI than children without comorbidities.

The follow-up periods within the different studies vary from weeks to years, so, at present, it is not possible to indicate an appropriate follow-up period for the evaluation of these patients. It is likely that further multicenter studies will clarify this issue in the future.

## 4. Conclusions and Future Perspectives

According to the literature, OSA is often a coexisting condition in children with laryngomalacia; these children should be evaluated endoscopically, with or without sedation, depending on their age. However, a child with persistent OSA after adenotonsillectomy with a history of stridor in infancy should always be evaluated endoscopically to exclude a pre-existing, unrecognized laryngomalacia. In the literature, DISE and PSG are suggested in the diagnostic work-up, although many centers often do not follow these indications.

This review highlights that (i) the management of patients with OSA and laryngomalacia is profoundly different in relation to age (</>2 years old) and that (ii) supraglottoplasty is the gold standard treatment for this disorder, even if the indication to this procedure is also related to patients age. In our opinion, further studies are necessary to evaluate more homogeneous samples of patients, preferably using the same methodologies. Additional progress will be necessary in the diagnostic work-up of OSA and laryngomalacia, particularly considering the polysomnographic features, in order to tailor the possible treatments further.

## Figures and Tables

**Table 1:** Etiologic theories.

Theory	Hypothesis	Evidence
Anatomic theory	Presence of supraglottic anatomic anomalies	Flaccid epiglottis, omega-shaped, posterior displacement of epiglottis short aryepiglottic fold, redundant arytenoideal mucosa
Cartilagineous theory	Alteration of the cartilaginous framework of larynx	Histological immaturity is seen in specimens of children who underwent supraglottoplasty
Neurologic theory	Possible lack of neuromotor coordination during breathing and swallowing.	Neurological comorbidities or prematurity are often associated. Weakness of laryngeal tone, presence of central sleep apneas. Possible spontaneous improvement during growth is reported.
Combination	Inflammation, airway pressure modification due to motor dysfunction could induce anatomical and cartilaginous modification of supraglottic anatomy.	GER seems to trigger all these factors (anatomic, cartilagineous, and neurological). Obesity.

**Table 3:** Treatment features within the included studies.

Autor/Year	N	Mean Age	Type of Instrument	Follow-Up Period	Post-Op Complication	Revision Surgery	Mean AHI Pre-Op	Mean AHI Post-Op
Valera 2006 [16]	7	6.8 mo.	Cold Knife	3 mo.	2 cases fail to extubation	2 cases tracheostomy	11.7	2.2
Zafereo 2008 [17]	10	4 mo.	Cold Knife	11 weeks	No	NR	12.2	4.2
O’Connor 2009 [18]	10	2.6 mo.	Cold Knife	3 mo.	1 case lung collapse	NR	42.7	4.5
Powitzky 2011 [19]	20	3.9 mo.	CO_2_ laser	9.5 mo.	NR	1 case supraglottoplasty 6 cases adenotonsillectomy	11.2 *	4.7 *
Digoy 2012 [26]	36	56 mo.	CO_2_ laser	3 mo.	NR	NR	13.3	4.1
Chan 2012 [3]	22	7.4 yrs.	CO_2_ laser	NR	No	NR	10.4	2.9
Mase 2015 [20]	9	17 mo.	CO_2_ laser Cold knife Microdebrider	155 days	NR	NR	23.5	4.8
Ching 2017 [21]	12	13.1 mo.	CO_2_ laser, Cold knife BRA	6 mo.	No	1 case tracheostomy	19.3	4
Cortes 2019 [22]	9	5.5 mo.	Cold knife	1 mo.	1 case foreign body reaction to epiglottopexy suture	No	34.87	9.44
Bhushan 2019 [23]	41	1.3 yrs.	CO_2_ laser	12.1 mo.	No	No	26.62	7.27
Verkest 2020 [24]	44	NR	Cold knife	3 mo.	4 cases, temporary feeding problems 2 cases fever/infection	NR	8.9 *	2.4 *
Casellas 2022 [25]	30	13.28 mo.	NR	NR	NR	NR	MiO 3.98 MoO: 6.8 SO: 29.6	MiO: 2.4 MoO: 2.2 SO: 5.4

Legend: NR: not reported; N: number of patients; mo.: months; yrs.: years; * Expressed as median; BRA: Bipolar Radiofrequency Ablation; MiO: Mild OSA; MoO: Moderate OSA; SO: Severe OSA.

## Data Availability

No new data were created or analyzed in this study. Data sharing is not applicable to this article, as this is a review paper.
